# How It Concerns Nurses

**Published:** 1920-11-27

**Authors:** 


### THE UNEMPLOYMENT INSURANCE ACT.

### How it Concerns Nurses.

On the occasion of the extraordinary general meeting of the College of Nursing on Saturday last, Miss Ford and Mr. Munro, of the Ministry of Labour, came by special invitation, at the end of the formal business, and delivered short addresses on the working of the new Unemployment Insurance Act and its requirements in the cases of nurses under the different kinds of employment. Sir Arthur Stanley remained in the chair. Much interest was shown by all present, and a number of questions were put to the speakers.

The Act came into force on November 8 last, and its provisions are compulsory, embracing workers who earn less than £250 a year, with one or two exceptions, such as agriculture and domestic service. The scheme covers those included under the National Health Insurance scheme. Exemption can be claimed by a person—otherwise eligible—who has a private income or pension of the annual value of £26 or more, or by a person dependent mainly on another person for his or her livelihood. Exemption does not relieve the employer of such person from his contribution. The contribution payable by the man is 4d. weekly, 4d. by the employer, and the State puts 2d. per week to the account. For women the employer gives 3½d., the employee 3d., and the State contributes 1⅔d. Smaller sums apply to persons of between sixteen and eighteen years of age.

With regard to benefits, for a man out of employment 15s. per week is paid; for a woman, 12s. This cannot be drawn for more than fifteen weeks in any one year; and not more than one week's benefit for every six weeks paid can be entertained. The first three days of unemployment are "waiting days"; payment of benefit starts on the fourth day. At the age of sixty years the nurse having paid not less than 500 contributions can claim a refund of the contributions paid—less any benefit received—with an added 2½ per cent.

One of the important exceptions provided for is where the nurse is employed under a local or other authority which is able to satisfy the Minister that the employed person is not subject to dismissal except on the ground of misconduct or unfitness to perform her duties, and in such cases the employing body must apply to the Minister to be so treated. To continue to be eligible for the relief, the person must remain willing to take work offered and be available for such; but where this involves travelling to a distant place, local possibilities would first be tried; and, failing success locally, reasonable time would be given before insisting on acceptance of the distant vacancy. No person can be regarded as out of work until she has registered the fact at an employment exchange.

One or two organisations—hospitals, etc.—have applied to the Ministry of Labour to be regarded, for the purposes of the Act, as a local authority, under Paragraph D.

With regard to the private nurse, acting "on her own," her contract is with the person she nurses, and that person must stamp her card with the contribution. A nurse sent out by an association, such as a co-operation, is held to be a servant of that body, and the association is liable. The duty of stamping rests with the employer, and it must be done weekly, being recoverable from the next wages due. No arrears owing to neglect can be so deducted. In the case of visiting nurses, the first person visited in the week is liable for the contribution, but an arrangement can be made whereby several employers can pay in rotation—a pooling arrangement.

With regard to the £250 limit, if a private nurse is paid three guineas a week, allowances for board, washing, etc., have been computed at two guineas, which brings the remuneration to more than the rate of £250 a year, hence such nurses will probably be regarded as exempt from the provisions of the Act. Value to the employee is considered, rather than cost to employer.

Miss Ford and Mr. Munro were cordially thanked by the Chairman at the conclusion of their lucid exposition of the Act from the nurses' point of view.

